# Computational investigations of physicochemical, pharmacokinetic, toxicological properties and molecular docking of betulinic acid, a constituent of *Corypha taliera* (Roxb.) with Phospholipase A2 (PLA2)

**DOI:** 10.1186/s12906-018-2116-x

**Published:** 2018-02-02

**Authors:** Mohammad Firoz Khan, Nusrat Nahar, Ridwan Bin Rashid, Akhtaruzzaman Chowdhury, Mohammad A. Rashid

**Affiliations:** 1 Department of Pharmacy, State University of Bangladesh, Dhaka, 1205 Bangladesh; 2 Department of Chemistry, Rajshahi University of Engineering and Technology, Rajshahi, Bangladesh; 3 Department of Pharmaceutical Chemistry, University of Dhaka, Dhaka, 1000 Bangladesh

**Keywords:** Physicochemical properties, Solvation free energy, Polarizability, Pharmacokinetic, Toxicology, Carcinogenic, Molecular docking

## Abstract

**Background:**

Betulinic acid (BA) is a natural triterpenoid compound and exhibits a wide range of biological and medicinal properties including anti-inflammatory activity. Therefore, this theoretical investigation is performed to evaluate (a) physicochemical properties such as acid dissociation constant (pKa), distribution coefficient (logD), partition coefficient (logP), aqueous solubility (logS), solvation free energy, dipole moment, polarizability, hyperpolarizability and different reactivity descriptors, (b) pharmacokinetic properties like human intestinal absorption (HIA), cellular permeability, skin permeability (P_Skin_), plasma protein binding (PPB), penetration of the blood brain barrier (BBB), (c) toxicological properties including mutagenicity, carcinogenicity, risk of inhibition of hERG gene and (d) molecular mechanism of anti-inflammatory action which will aid the development of analytical method and the synthesis of BA derivatives.

**Methods:**

The physicochemical properties were calculated using MarvinSketch 15.6.29 and Gaussian 09 software package. The pharmacokinetic and toxicological properties were calculated on online server PreADMET. Further, the molecular docking study was conducted on AutoDock vina in PyRx 0.8.

**Results:**

The aqueous solubility increased with increasing pH due to the ionization of BA leading to decrease in distribution coefficient. The solvation energies in water, dimethyl sulfoxide (DMSO), acetonitrile, *n*-octanol, chloroform and carbon tetrachloride were − 41.74 kJ/mol, − 53.80 kJ/mol, − 66.27 kJ/mol, − 69.64 kJ/mol, − 65.96 kJ/mol and − 60.13 kJ/mol, respectively. From the results of polarizability and softness, it was clear that BA is less stable and hence, kinetically more reactive in water. BA demonstrated good human intestinal absorption (HIA) and moderate cellular permeability. Further, BA also exhibited positive CNS activity due to high permeability through BBB. The toxicological study revealed that BA was a mutagenic compound but noncarcinogenic in mice model. Moreover, molecular docking study of BA with PLA2 revealed that BA interacts with GLY22 & GLY29 through hydrogen bond formation and LEU2, PHE5, HIS6, ALA17, ALA18, HIS47 and TYR51 through different types of hydrophobic interactions. The binding affinity of BA was − 41.00 kJ/mol which is comparable to the binding affinity of potent inhibitor 6-Phenyl-4(R)-(7-Phenyl-heptanoylamino)-hexanoic acid (BR4) (− 33.89 kJ/mol).

**Conclusions:**

Our computed properties may assist the development of analytical method to assay BA or to develop BA derivatives with better pharmacokinetic and toxicological profile.

## Background

**Fig. 1:**
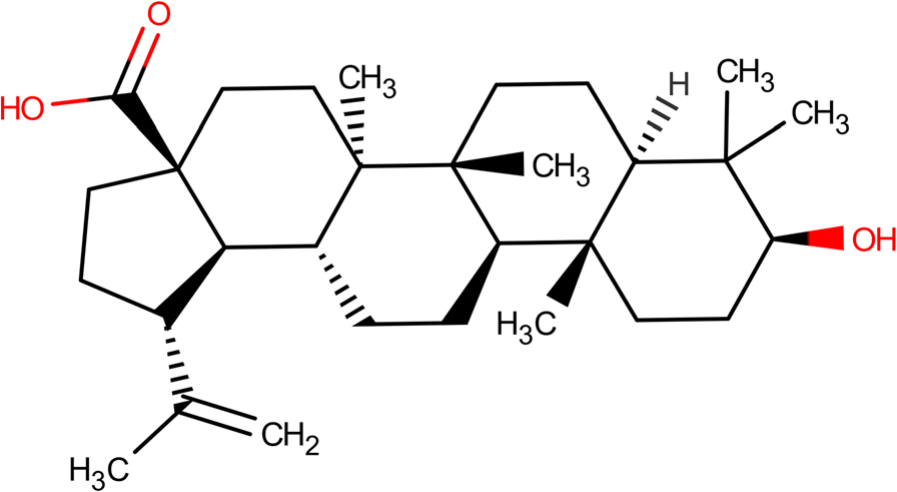
Structure of betulinic acid

Betulinic acid (BA), (3β-hydroxy-lup-20(29)-en-28-oic acid) (Fig. 1) is a natural pentacyclic lupane type triterpenoid compound and exhibits a wide range of biological and medicinal properties such as antivenom [1], anti-HIV [2, 3], antibacterial [2], antimalarial [4], anti-inflammatory [5–7] anthelmintic [8], antinociceptive [9], anti-HSV-1 [10, 11] and anticancer activities [12–14]. It is abundantly distributed throughout the plant kingdom [15]. The birch tree (*Betula* spp., Betulaceae) is one of the most widely reported sources of BA which can be obtained in considerable quantities [16, 17]. BA can also be isolated from various sources including *Corypha taliera* [18], *Ziziphus* spp. (Rhamnaceae) [19, 20], *Syzygium* spp. (Myrtaceae) [21], *Diospyros* spp. (Ebenaceae) [22, 23] and *Paeonia* spp. (Paeoniaceae) [24]. Betulin, the reduced form of BA, was one of the first natural products to be isolated from the bark of the white birch, *Betula alba* [25].

BA was shown to exert its diverse pharmacological activities with variable median inhibitory concentrations (IC_50_) such as anticancer activity by inhibiting DNA Topoisomerases (Topos) II at IC_50_ of 56.12 μM [26], anti-HIV activity at IC_50_ of 23.65 μM [27], anti-malarial activity at IC_50_ of 56.71 μM [28], anti-fungal property at IC_50_ of 14.23 μM [29], anti-protozoal activity at IC_50_ of 50 μM [30].

BA also inhibited DNA polymerase beta at IC_50_ of 30.65 μM [31], protein tyrosine phosphatase 1B (PTP1B) at IC_50_ value of 1.5 μM [32], inhibited *T. brucei* GAPDH COX-1, COX-2 and LT formation in vitro with IC_50_ values of 240 μM [33], > 125 μM, > 125 μM and 102.2 μM [34], respectively. BA displayed potent anti-inflammatory activity by inhibiting Phospholipase A2 (PLA2) and showed 30% and 40% inhibition of PLA2 at concentrations of approximately 2.5 and 5 μM, respectively [5].

Earlier studies showed that BA exhibited anti-inflammatory activity by inhibiting TNF- alpha and increasing the production of anti-inflammatory cytokine IL-10 [35]. Kim and colleagues demonstrated that betulinic acid exerted its anti-inflammatory activity by inhibiting the nuclear factor-kappa beta pathway, production of nitric oxide (NO), prostaglandin E2 (PGE2), tumor necrosis factor- alpha, interleukin-6 (IL-6), and interleukin-1 beta levels [36]. Anti-inflammatory activity was also seen in encephalitogenic T cells where betulinic acid inhibited IL-17 and IFN-γ production [37]. Arachidonoyl trifluoromethyl ketone, bromoenol lactone, varespladib, varespladib methyl, ecopladib, efipladib, giripladib, pyrrophenone, pyrroxyphene, FPL67047XX, inhibitor 22, amide 23 (GK115), 2-oxoamides, 1,3-disubstituted propan-2-ones are all synthetic phospholipase A2 inhibitors that have been used for clinical cases [38]. Among natural compounds, extracts of curcumin, *Ginkgo biloba* and *Centella asiatica* have demonstrated to be phospholipase A2 inhibitors and have even been used to treat neurological disorders characterized by neuroinflammation [38]. Bernard and colleagues experimentally proved that betulin and betulinic acid were potent phospholipase A2 inhibitors [5].

Phospholipase A2 (PLA2) hydrolyzes the membrane glycerophospholipids and releases arachidonic acid eventually leading to the production of pro-inflammatory mediators such as leukotrienes, prostaglandins, platelet activating factors (PAF) [39]. Thus, inhibiting PLA2 activity and hence regulating the production of pro-inflammatory mediators for the development of therapeutics against inflammatory diseases [40, 41] is a plausible approach. However, the PLA2 exists in two isoforms [40, 42], which includes the low molecular weight (14 kDa) Ca^+ 2^ dependent extracellular PLA2 found in mammalian pancreases, several snake venoms, human platelets, human placentas, rheumatoid synovial fluids [43] and the high molecular weight (85 kDa) cytosolic PLA2 [44]. Convincing evidence suggests that the14 kDa PLA2 might be a potential target for the modulation of inflammatory diseases [40], here we have reported the molecular docking study of BA against human secretory (14 kDa) PLA2 (1KQU) to explore the molecular basis of anti-inflammatory action of BA.

Few computational and theoretical studies of BA have been reported. BA associates with human serum albumin via hydrogen bond with PHE206 & GLU354 and hydrophobic interactions with PHE206, ARG209, ALA210, ALA213, LEU327, GLY328, LEU331, ALA350 and LYS351, in the sub-domain IIA and IIB of the large hydrophobic cavity [45].

In this investigation, computational studies have been carried out to evaluate (a) physical and chemical properties such as acid dissociation constant (pKa), distribution coefficient (logD), partition coefficient (logP), aqueous solubility (logS), solvation free energy, dipole moment, polarizability, hyperpolarizability and different reactivity descriptors (chemical hardness, softness, chemical potential, electronegativity, electrophilicity index), (b) pharmacokinetic properties like human intestinal absorption (HIA), cellular permeability using Caco-2 cell model, skin permeability (P_Skin_), plasma protein binding (PPB), penetration of the blood brain barrier (BBB), (c) toxicological properties including mutagenicity, carcinogenicity, risk of inhibition of human ether-a-go-go-related (hERG) gene and (d) molecular mechanism of anti-inflammatory action of BA. The purpose of this study was to investigate the physicochemical, pharmacokinetic and toxicological properties and to correlate the calculated physicochemical properties with the absorption and distribution profile of BA. These in silico investigations will provide an insight for the development of analytical method to assay BA [46] or to develop BA derivatives with better pharmacokinetic & toxicological profile and having more potent anti-inflammatory activity.

## Methods

### Computational methods

The acid dissociation constant (pKa), distribution coefficient (logD), partition coefficient (logP) and aqueous solubility (logS) of betulinic acid (BA) over the pH range of 0.0 to 14.0at 298 K were calculated using MarvinSketch 15.6.29 (ChemAxon, Hungary) (http://www.chemaxon.com). The consensus logP method was applied to calculate distribution coefficient of the molecule.

The rapid progress in computational methods such as Gaussian family of methods (G1, G2, G2MP2, G3) and complete basis set extrapolation (CBS) method enable researchers to perform highly sophisticated calculations of enthalpies and thermochemical properties with minor errors in comparison to experimental data [47–51]. However, these methods are computationally very expensive. An alternative to these high cost calculations is the use of density functional theory (DFT) methods. Previous report revealed that [52] calculation of geometries (bond length, bond angle and dihedral angle) and thermochemical properties using DFT/B3LYP level of theory showed better agreement with the experiments. So, in the current investigation, the calculation of solvation free energy, dipole moment, polarizability, hyperpolarizability and global reactivity descriptor properties such as the chemical hardness, softness, chemical potential, electronegativity, electrophilicity index were conducted in gas phase and in different solvents namely water, dimethyl sulfoxide (DMSO), acetonitrile, *n*-octanol, chloroform and carbon tetrachloride with the B3LYP/6-31G(d) level of theory implemented in Gaussian 09 software package [53]. All calculations were conducted using the optimized geometry which was confirmed by the absence of imaginary frequency in the lowest energy state of the molecule. The Solvation Model on Density (SMD) [54] was used for all calculations involving the solvents. All calculations involving solvation were performed using the optimized solution-phase structures.

The pharmacokinetic and toxicological properties were calculated using online server PreADMET (https://preadmet.bmdrc.kr/). The pharmacokinetic properties such as human intestinal absorption (HIA), in vitro cellular permeability using Caco-2 cell model, skin permeability (P_Skin_), plasma protein binding (PPB) and penetration of the blood-brain barrier (BBB), interaction with P-glycoprotein (Pgp) and metabolism (both phase I and phase II) were calculated and predicted. In addition, BA was virtually screened to evaluate toxicological properties such as mutagenicity, carcinogenicity and risk of inhibition of human ether-a-go-go-related (hERG) gene.

### Molecular docking study

#### Preparation of target protein X-ray structure

The first step of docking study is to select an appropriate X-ray crystal structure of the target protein that is already bound to its known ligand. This is because during docking, software searches complementary binding site/(s) for ligand within the search space of the target protein. That’s why ligand bound conformation of protein structure is the prerequisite to perform molecular docking. Moreover, the structure should be solved with a reasonable accuracy which is reflected in the statistics for data collection and processing of X-ray crystal structure. In addition, refinement statistics such as R_work_/R_free_, RMS deviation from ideality (bonds and angles) and validation parameters including Ramachandran outliers, rotamer outliers, bad bond count or bad angle count etc. also indicate the quality of the model structure. In our current investigation, we have selected the crystal structure of secretory PLA2 complexed with 6-Phenyl-4(R)-(7-Phenyl-heptanoylamino)-hexanoic acid (BR4) (PDB code: 1KQU) [55] since the structure was solved at 2.1 Å resolution with R_work_/R_free_ of 0.209/0.240 and the validation parameters indicates good quality of the model structure. Water molecules and hetero atoms were then removed from the protein using PyMOL (Version 1.7.4.4, Schrödinger). Energy minimization was performed by applying YASARA force field level of theory in YASARA Energy Minimization Server (http://www.yasara.org/minimizationserver.htm).

#### Preparation of ligands

The structure of the BR4 and BA were drawn and optimized in Gaussian 09 [53] with density functional theory (DFT) at the B3LYP/6-31G(d) level of theory. The optimized structures of ligands (BR4 and BA) were saved in PDB format for docking study.

#### Protein-ligand docking

Several virtual screening tools are available to perform molecular docking such as BINDSURF, METADOCK, Lead Finder, FlexScreen, AutoDock vina etc. Scoring functions implemented in these softwares calculate the protein-ligand interactions energy by computing electrostatic, Van der Waals and hydrogen bonding terms [56–60]. In addition to these scoring functions AutoDock vina also calculates the hydrophobic interactions [60] which is crucial for calculating the interaction energies of protein and hydrophobic ligand. Since BA is hydrophobic in nature, we used AutoDock vina to explore not only the electrostatic, Van der Waals and hydrogen bonding but also the hydrophobic interactions.

**Fig. 2:**
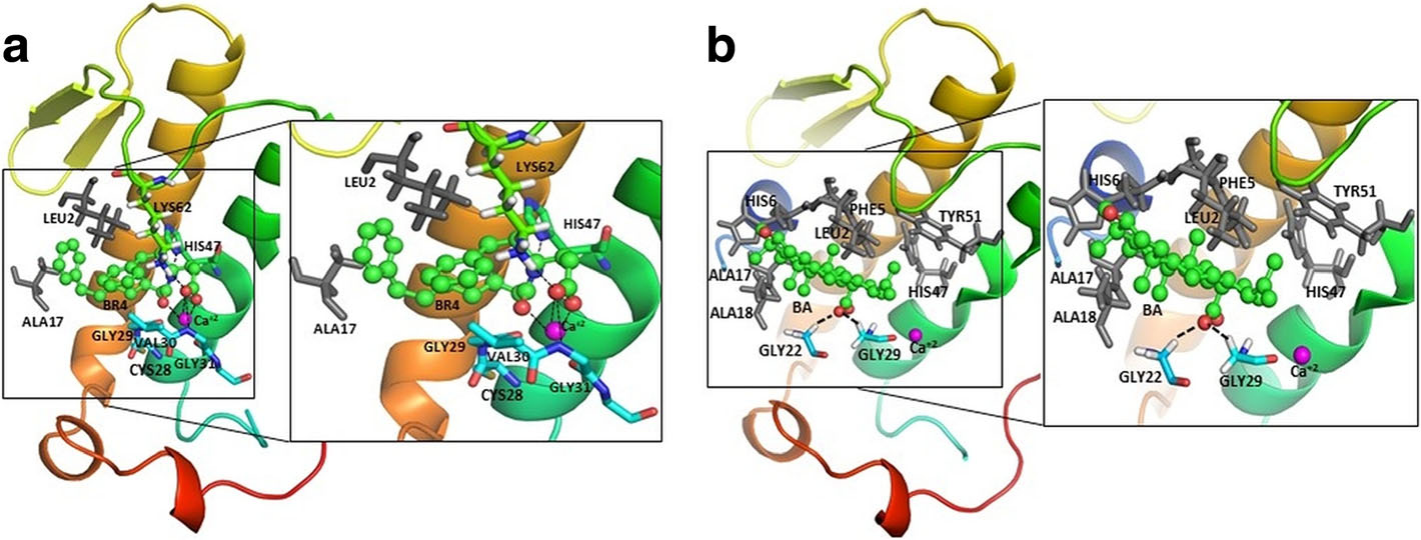
Interaction of (**a**). BR4 (green) and (**b**). BA (green) with PLA2 visualized in pymol. The calcium ion (Ca^+ 2^) is presented as magenta color sphere. Black dash indicates H bonding. Residues involve in hydrophobic interactions are shown in gray color

**Fig. 3:**
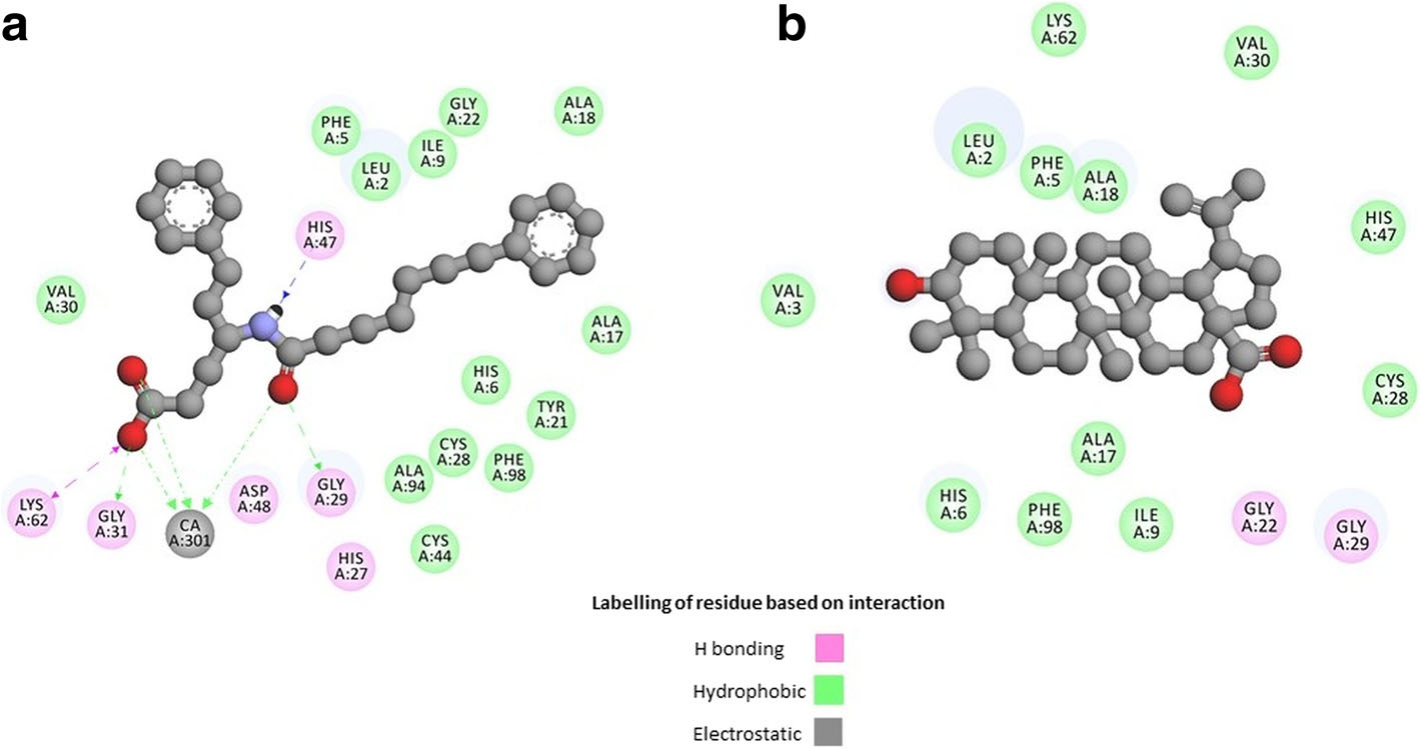
Interaction of (**a**). BR4 (gray) and (**b**). BA (gray) with PLA2 visualized in Drug Discovery studio

The docking of target protein with the ligands was conducted using AutoDock vina [60] in PyRx 0.8 (https://pyrx.sourceforge.io/). Docking study was performed to get a set of possible conformations and orientations for the ligand at the binding site. Using PyRx software, the PLA2 and ligands were prepared after which docking was conducted using a grid whose center was (56.5961, 34.0180, 42.4808) and the dimensions were (25.00, 25.00, 25.00) Å. During the docking analysis, the drug molecules were flexible and the macromolecule was kept rigid. Ligand displaying the lowest binding affinity and ability to bind in the binding pocket of protein was chosen as the best conformation. The interactions of different residues of protein with ligands such as hydrogen bonds, electrostatic interactions, hydrophobic interactions and bond distances were analyzed by PyMOL and Discovery Studio visualizer v4.0.100.13345 (Figs. 2 and 3).

**Fig. 4:**
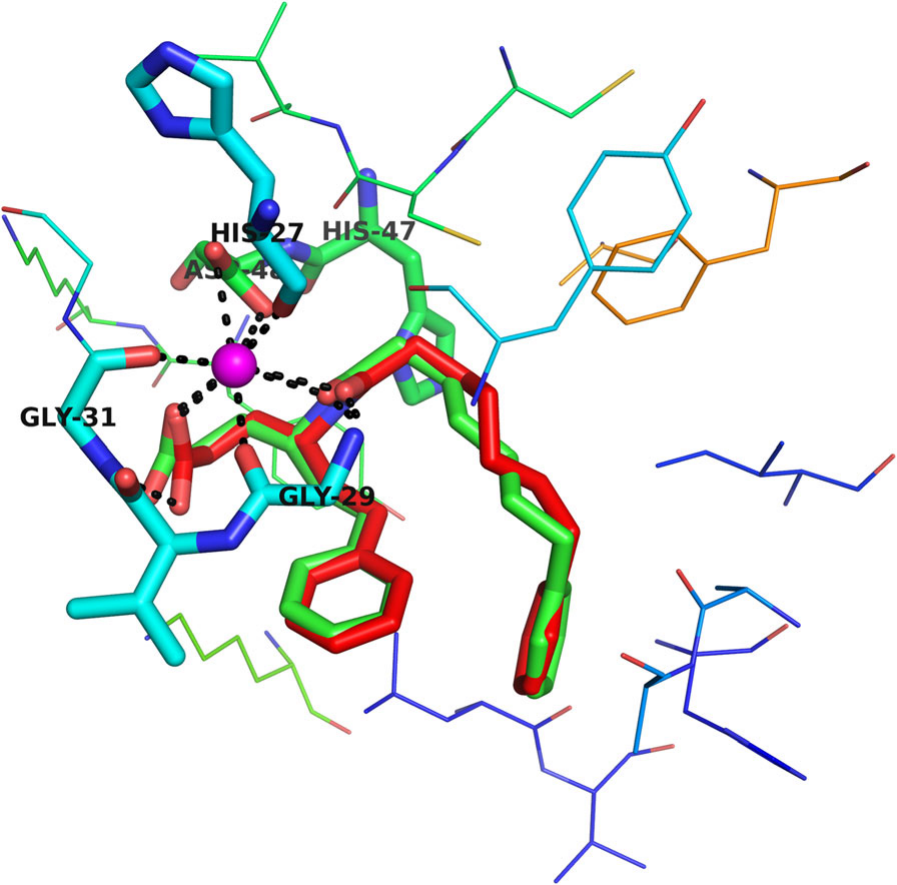
The superimposition of the best docking structure with the X-ray structure visualized in PyMOL; () experimental ligand, () docked ligand

Before docking of BA, the protocol was validated by re-docking BR4 into the binding pocket of 1KQU to get the docked pose and root mean square deviation (RMSD). The result revealed that the first pose of BR4 almost superimposed (RMSD of heavy atoms constituting the backbone of molecule is 0.5271) with the experimental crystal structure of BR4 (Fig. 4). Thus, the docking method has reasonable accuracy and reproducibility and can be used for further docking experiments.

## Results and discussion

### Calculation of pKa

**Fig. 5:**
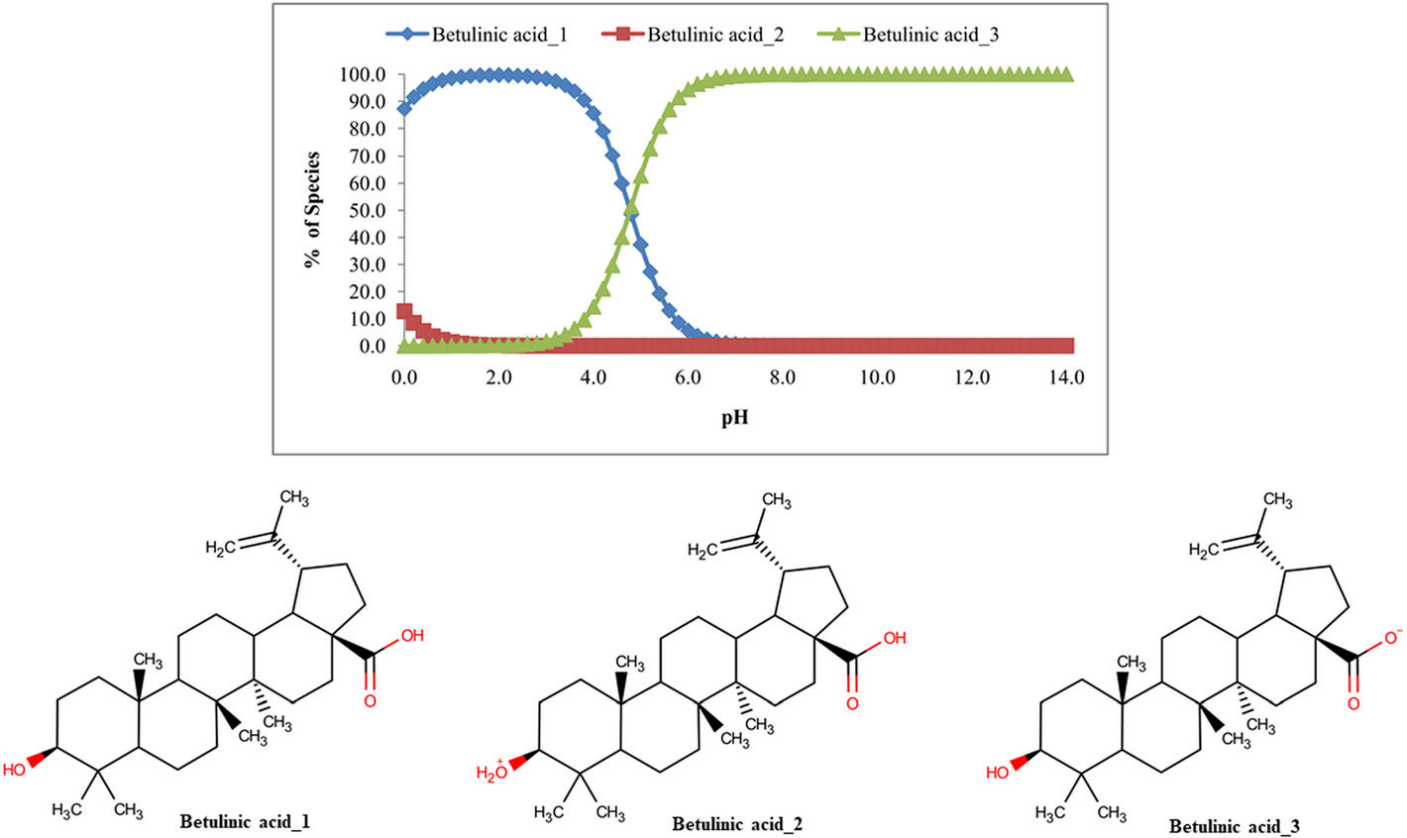
Microspecies distribution (%) of betulinic acid

The calculation of pKa revealed that BA has two ionized species along with the unionized form (Fig. 5). It is clear from the figure that non-ionized form (Betulinic acid_1) predominates over the pH range of 0.0 to 4.6 whereas the betulinate form (Betulinic acid_3) dominates from pH 4.8 to 14.0. However, small amount of Betulinic acid_2 was present over the pH of 0.0 to 2.4.

### Distribution (logD) coefficient and partition (logP) coefficient

**Fig. 6:**
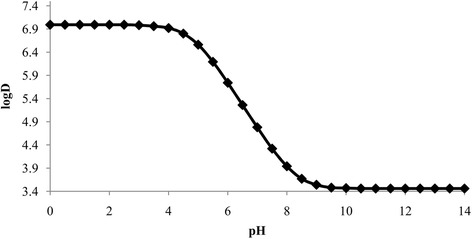
Distribution coefficient (logD) of betulinic acid over the pH range of 0.0 to 14.0

The logD was calculated using consensus method implemented in MarvinSketch 15.6.29. The logD vs pH of BA is presented in Fig. 6. The logD value decreases with increasing pH of the solution suggesting that the prevalence of the unionized form of BA decreases and the ionized form (betulinate) increases with increasing pH of the solution. This result is in accordance with the calculation of pKa.

The partition coefficient (logP) of BA was also computed and presented in Table 1. The logP calculated in MarvinSketch 15.6.29 was 6.64 whereas the experimental logP of BA were reported as 6.61 [61] and 6.85 [62].

**Table 1:** Comparison of partition coefficient of betulinic acid

	logP
MarvinSketch 15.6.29	6.64
Experimental (40)	6.61
Experimental (41)	6.85

### Aqueous solubility (logS)

**Fig. 7:**
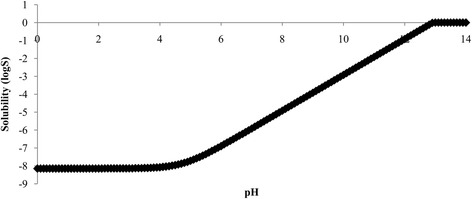
Solubility (logS) of betulinic acid over the pH range of 0.0 to 14.0

The aqueous solubility of BA in terms of logS is presented in Fig. 7. The figure indicates that the solubility increases with increasing pH of the solution. However, the intrinsic solubility of BA was found − 7.34 (0.000000046 mol/L) and the aqueous solubility at pH 7.4 was − 4.79 (0.000016 mol/L) indicating that BA is practically insoluble in water.

### Solvation free energy

The solvation free energies of BA were calculated with the SMD model [54] The values are summarized in Table 2. The solvation energies of BA in water, DMSO, acetonitrile, *n*-octanol, chloroform and carbontetrachloride were − 41.74 kJ/mol, − 53.80 kJ/mol, − 66.27 kJ/mol, − 69.64 kJ/mol, − 65.96 kJ/mol and − 60.13 kJ/mol, respectively. It could therefore be concluded that solvation free energy increases with decreasing polarity of polar nonprotic solvent (DMSO and acetonitrile). The highest value of solvation energy was in non-polar protic solvent (*n*-octanol). The free energy decreases with decreasing polarity of non-polar aprotic solvent (chloroform and carbon tetrachloride). Thus, it could be stated that BA associates with non-polar portion (hydrocarbon chain of *n*-octanol) via hydrophobic interactions. Electrostatic interactions might arise due to the association of the carboxyl group of BA with the polar portion (hydroxyl group of *n*-octanol) (Table 5).

**Table 2:** Solvation free energy (kJ/mol) of betulinic acid in different solvents with SMD

Medium (dielectric constant)	Solvation free energy (kJ/mol)
Water (78.3)	−41.74
DMSO (46.8)	−53.80
Acetonitrile (35.7)	−66.27
*n*-Octanol (9.9)	− 69.64
Chloroform (4.7)	−65.96
Carbon tetrachloride (2.2)	−60.13

### Dipole moment

**Fig. 8:**
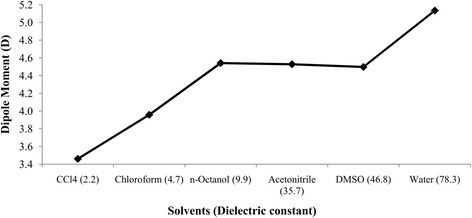
Effect of solvent polarity on dipole moment (D) of betulinic acid

The dipole moment of BA is found to be higher in different solvents than that of the gas phase. Table 3 presents the dipole moments computed in the gas phase and different solvents (water, DMSO, acetonitrile, *n*-octanol, chloroform and carbon tetrachloride) at the B3LYP level of theory with 6-31G(d) basis set using SMD solvation model. The dipole moments were 2.98D, 5.13D, 4.50D, 4.53D, 4.54D, 3.96D and 3.46D in the gas phase, water, DMSO, acetonitrile, *n*-octanol, chloroform and carbon tetrachloride, respectively. Therefore, increasing dielectric constant of the solvent is accompanied by a gradual increase in the dipole moment. In other words, the dipole moment increases with the increasing polarity of the solvent (Fig. 8).

**Table 3:** Dipole moment (Debye, (D)) of betulinic acid in gas phase and in different solvents using SMD

Medium (dielectric constant)	Dipole Moment (D)
Gas	2.98
Water (78.3)	5.13
DMSO (46.8)	4.50
Acetonitrile (35.7)	4.53
n-Octanol (9.9)	4.54
Chloroform (4.7)	3.96
Carbon tetrachloride (2.2)	3.46

### Polarizability and first order hyperpolarizability

The polarizability(α) of BA was calculated using the following equation:

$$ \alpha =\frac{1}{3}\ \left({\alpha}_{xx}+{\alpha}_{yy}+{\alpha}_{zz}\right) $$

The quantities α_xx_, α_yy_ and α_zz_ are known as principal values of polarizability tensor.

**Fig. 9:**
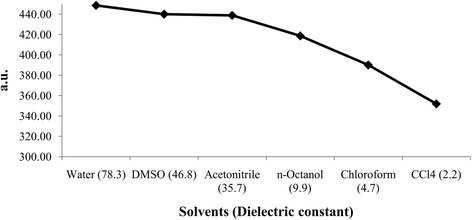
Effect of solvent polarity on polarizability of betulinic acid

The calculated polarizability of BA in different solvents is presented in Table 4, which showed that polarizability (*α*_*tot*_) ranged from 351.90 to 448.67 a.u. The plot of polarizability vs. solvent is shown in Fig. 9. It is clear from the figure that the polarizability gradually increases when going from lower to higher dielectric constant. This suggests that the kinetic reactivity of BA increases with increasing polarity of the solvent [63].

**Table 4:** Effect of solvent polarity on polarizability (a.u.) and first order hyperpolarizability (a,u)

Medium (dielectric constant)	α_xx_	α_yy_	α_zz_	α_tot_	β_x_	β_y_	β_z_	β_tot_
Gas Phase	355.73	300.52	260.89	305.72	103.92	−121.26	−37.58	164.06
Water (78.3)	447.54	459.46	439.01	448.67	154.68	− 170.92	−87.52	246.57
DMSO (46.8)	441.82	450.17	428.25	440.08	132.92	− 155.98	−68.48	216.07
Acetonitrile (35.7)	442.00	448.66	425.71	438.79	130.61	−155.78	−70.89	215.30
*n*-Octanol (9.9)	434.46	426.93	394.48	418.62	139.39	− 157.93	−74.51	223.43
Chloroform (4.7)	418.61	395.58	356.17	390.12	124.74	− 146.22	−58.57	200.93
Carbon tetrachloride (2.2)	393.70	352.38	309.63	351.90	117.66	− 134.29	−46.43	184.48

The first order hyperpolarizability (*β*_*tot*_) is the measure of the nonlinear optical activity and can be calculated using the following equation:

$$ {\beta}_{tot}={\left({\beta}_x^2+{\beta}_y^2+{\beta}_z^2\right)}^{\raisebox{1ex}{$1$}\!\left/ \!\raisebox{-1ex}{$2$}\right.} $$

Where,

$$ {\displaystyle \begin{array}{l}\begin{array}{l}\ {\beta}_x={\beta}_{xxx}+{\beta}_{xyy}+{\beta}_{xzz}\\ {}{\beta}_y={\beta}_{yyy}+{\beta}_{xxy}+{\beta}_{yzz}\end{array}\\ {}{\beta}_z={\beta}_{zzz}+{\beta}_{xxz}+{\beta}_{yyz}\end{array}} $$

**Fig. 10:**
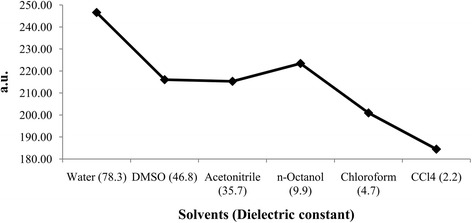
Effect of solvent polarity on first order hyperpolarizability of betulinic acid

The *β*_*tot*_ for different solvents was listed in Table 4, which displayed that the hyperpolarizability in different solvents ranged from 184.48 to 246.57 a.u. Moreover, the hyperpolarizability increases as the dielectric constant increases except for non-polar protic *n*-octanol where the hyperpolarizability is higher than that of acetonitrile and DMSO (Fig. 10).

### Global reactivity descriptors

**Fig. 11:**
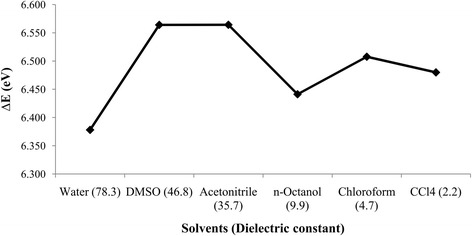
Effect of solvent polarity on HOMO-LUMO energy gap of betulinic acid

The highest occupied molecular orbital-lowest unoccupied molecular orbital (HOMO-LUMO) energy gap represents the stability or reactivity of molecules. Higher energy gap indicates greater stability and vice versa. The values of HOMO-LUMO energy gap in various solvents are presented in Table 5 and their trend is shown in Fig. 11. From Fig. 11, it is clear that the HOMO-LUMO energy gap is the highest in DMSO and acetonitrile, indicating greater stability of BA in these solvents, however lowest HOMO-LUMO energy gap was found in water meaning the titled molecule is less stable and hence, more reactive in water which is in agreement with calculated polarizability, chemical hardness and softness. The Table 5 and Fig. 11 suggest that the molecule is stabilized with decreasing polarity of the solvent i.e. the molecule is less likely to be kinetically reactive.

**Table 5:** Molecular Orbital Energy (eV) **(**HOMO and LUMO) of betulinic acid in different solvents with SMD

Medium (dielectric constant)	Molecular Orbital Energy (eV)		
	*HOMO*	*LUMO*	*∆E*
Gas Phase	−6.305	0.133	6.438
Water (78.3)	−6.262	0.116	6.378
DMSO (46.8)	−6.243	0.321	6.564
Acetonitrile (35.7)	−6.244	0.321	6.565
*n*-Octanol (9.9)	−6.241	0.201	6.441
Chloroform (4.7)	−6.222	0.286	6.508
Carbon tetrachloride (2.2)	−6.229	0.251	6.480

The global chemical reactivity descriptors such as softness, hardness, chemical potential and electrophilicity index can be calculated from the HOMO-LUMO energy gap of a molecule [64–68]. Using Koopman’s theorem for closed-shell molecules the hardness (η), chemical potential(μ), electronegativity (χ) and softness (S) are calculated according to the following equation:

$$ \eta =\frac{I-A}{2} $$

$$ \mu =-\frac{I+A}{2} $$

$$ \chi =\frac{I+A}{2} $$

$$ S=\frac{1}{\eta } $$

Where

Ionization potential, I = -E_HOMO_

Electron affinity, A = -E_LUMO_

The electrophilicity index (ω) is calculated according to equation derived (by Parr et al., [68]) [58] as follows:

$$ \omega =\frac{\mu^2}{2\eta } $$

The molecular properties of BA in different solvents are presented in Table 6. No systematic trend was found in the case of chemical hardness & softness, chemical potential, electronegativity and electrophilicity index. However, the highest chemical softness, electronegativity and electrophilicity index was found in water (polar protic) followed by *n*-octanol (non-polar aprotic) and carbon tetrachloride (non-polar aprotic).

**Table 6:** Effect of solvent polarity on molecular properties of betulinic acid

Medium (dielectric constant)	Chemical hardness (η)	Softness (S)	Chemical potential (μ)	Electronegativity (χ)	Electrophilicity index (ω)
Gas Phase	3.219	0.311	−3.086	3.086	1.479
Carbon tetrachloride (2.2)	3.240	0.309	−2.989	2.989	1.379
Chloroform (4.7)	3.254	0.307	−2.968	2.968	1.354
*n*-Octanol (9.9)	3.221	0.310	−3.020	3.020	1.416
Acetonitrile (35.7)	3.282	0.305	−2.962	2.962	1.336
DMSO (46.8)	3.282	0.305	−2.961	2.961	1.336
Water (78.3)	3.189	0.314	−3.073	3.073	1.480

### Pharmacokinetic study

Pharmacokinetic studies such as absorption, distribution and metabolism of BA was done by using the web based application PreADMET (https://preadmet.bmdrc.kr/). The calculated absorption, distribution and metabolism parameters are presented in Table 7.

**Table 7:** Absorption, distribution and metabolism of betulinic acid

Absorption			
HIA (%)	P_Caco-2_ (nm/s)		P_skin_
96.00	21.86		−2.11
Distribution			
PPB (%)	C_brain_/C_blood_	P-Glycoprotein (Inhibition)	P-Glycoprotein (Substrate)
100.00	8.20	Inhibitor	Substrate
Metabolism			
Phase I		Phase II	
*Enzyme*	*Inhibitor/Substrate*	*Enzyme*	*Substrate/Non-substrate*
Cytochrome P450 2C19 (Inhibitor)	Non	UDP-glucuronosyltransferase (UGT)	Non-substrate
Cytochrome P450 2C9 (Inhibitor)	Inhibitor	Sulfotransferase (SULT)	Non-substrate
Cytochrome P450 2D6 (Inhibitor)	Non		
Cytochrome P450 2D6 (substrate)	Non		
Cytochrome P450 3A4 ((Inhibitor)	Inhibitor		
Cytochrome P450 3A4 (substrate)	Substrate		

The calculated human intestinal absorption HIA of BA (Table 7) was found to be 95.996% which suggests that BA is well absorbed through the intestinal cell [69]. BA was moderately permeable since values for absorption through Caco-2 cell (P_Caco-2_) was 21.86 [70]. The skin permeability (P_Skin_) is a vital parameter for assessment of drugs and chemical that might require transdermal administration [71]. BA was found to be impermeable through skin since a calculated P_Skin_ was − 2.11 encountered which proves BA is not permeable through the skin.

The distribution properties were assessed by measuring the brain to blood partitioning (C_brain_/C_blood_) and plasma protein binding (PPB). Generally, compounds with more than 90% of PPB are classified as strongly bound chemicals whereas less than 90% are weakly bound chemicals (https://preadmet.bmdrc.kr/adme-prediction/). The calculated value of PPB for BA was 100.00% indicating BA is a strongly bound chemical and hence, the free form of BA will be less available in the systematic circulation. Based on C_brain_/C_blood_ ratio all chemicals fall under three categories namely high absorption to CNS (C_brain_/C_blood_ value more than 2.0), moderate absorption to CNS (C_brain_/C_blood_ value within 2.0–0.1) and low absorption to CNS (C_brain_/C_blood_ value less than 0.1) [72]. The ratio of C_brain_/C_blood_ (8.20) suggests high absorption of BA to CNS indicating higher ability to cross blood brain barrier (BBB).

The exchangeable fraction (sum of unbound fraction and fraction dissociated from the protein) of drug determines the amount of drug that will penetrate the BBB [73]. Besides, Pardridge and colleagues have reported the permeation of protein-bound compounds through the BBB [74–76]. Since BA is a strongly protein bound chemical (100%) so it could be assumed that BA will penetrate the BBB either due to the fraction dissociated from the plasma protein or as a protein-bound form or both.

P-glycoprotein (Pgp) is the product of the multi drug resistance (MDR) gene and an ATP dependent efflux transporter that affects the absorption, distribution and excretion of clinically important drugs [77]. Over-expression of this protein, which may result in MDR is a major cause of the failure of cancer chemotherapy, and decreased efficacy of antibiotics [78, 79]. The prediction of Pgp substrates, which facilitates early identification and elimination of drug candidates of low efficacy or high potential of MDR [80, 81]. Identifying molecules that interact with Pgp transporters is important for drug discovery, but it is commonly determined through laborious in vitro and in vivo studies [82]. Computational classification model can be used to screen molecules and predict the likeliness to be substrate for Pgp [82]. The in silico screening revealed that BA is a dual inhibitor and substrate for Pgp like quinidine [83]. This is because due to complex modulatory interactions with the Pgp which make BA to function as combination of substrate and inhibitor [83].

The computed metabolism of BA (Table 7) displayed that it is an inhibitor of CYP2C9 and a dual inhibitor and substrate for CYP3A4 due to complex modulation of CYP3A4 in phase I reaction. In phase II reaction BA is neither a substrate for UDP-glucuronosyltransferase (UGT) nor sulfotransferase (SULT).

### Toxicological study

The PreADMET server (https://preadmet.bmdrc.kr/) was used to evaluate the carcinogenicity of BA. It was found that although BA is mutagenic but it demonstrated noncarcinogenicity in mice. On the other hand, BA is unlikely to be an inhibitor of human ether-a-go-go-related (hERG) gene (low risk). Inhibition of the hERG gene has been linked to long QT syndrome [84]. The results have been in summarized in Table 8.

**Table 8:** Toxicological properties of betulinic acid

Mutagenicity (Ames test)	Carcinogenicity in Mouse	hERG (Inhibition)
Mutagenic	Negative	Low risk

### Protein optimization

YASARA Energy Minimization Server (http://www.yasara.org/minimizationserver.htm) [85] was used to optimize the structure of PLA2. This server utilizes a new partly knowledge-based all atom force field derived from Amber, whose parameters have been optimized to attain the protein structure as close as to its native structure with maximum accuracy [85]. The simulation was performed in a water sphere containing ions.

The structure validation Z-scores and force field energies before and after the minimization are displayed in Table 9. The energies of the structure before and after optimization were − 68,549.6 and − 83,729.2 kJ/mol, respectively. This indicates that the protein structure was stabilized by an amount of − 15,179.6 kJ/mol. In addition, the structure validation Z-scores of before and after optimization was − 0.1 and 1.15 indicating good optimization of the target protein. The structure of PLA2 after energy minimization in a water sphere is presented in Fig. 11. PyMOL (Version 1.7.4.4, Schrödinger) was used to evaluate the root mean square deviation (RMSD) between the initial and final structure of PLA2 which was found to be 0.363.

**Table 9:** Optimization parameter of phospholipase A2 (1KQU)

	Force field energy (kJ/mol)	Structure validation Z-score	RMSD of initial and final structure
Before optimization	−68,549.6	−0.1	0.363
After optimization	−83,729.2	1.15	
Difference (∆)	−15,179.6	1.25	

### Molecular docking

Molecular docking of BA was carried out with human PLA2 using AutoDock Vina, to identify and understand the binding mode of BA and the intermolecular interactions with the target protein. All reported PLA2 inhibitors possess three key enzyme binding components such as Ca^+ 2^ binding oxygen atom, a HIS47 binding H-bond donor and a hydrophobic component that bind the active site of the enzyme [55]. So, PLA2 inhibitors should form electrostatic interactions with Ca^+ 2^ ion, H bond formation with the catalytic HIS47 and different types of hydrophobic interactions with hydrophobic residues which line the active site cavity of the enzyme. In addition, the inhibitors should interact and displace the HIS6; a unique residue to human PLA2 enzyme [55]. From our docking study, it was found that BA interacts with PLA2 at a binding affinity of − 41.00 kJ/mol whereas for BR4 it was − 33.89 kJ/mol. BA lies deep within the active site activity and makes numerous close contacts (< 5.4 Å) with the enzyme through hydrogen bond formation with GLY22 & GLY29 and hydrophobic interactions with the LEU2, PHE5, HIS6, ALA17, ALA18, TYR51 and the catalytic residue HIS47 (Figs. 2 and 3 and Table 10). However, no interactions of BA with calcium were observed as it was found in case of bovine PLA2 [5]. Therefore, BA has the ability to interact with the catalytic residue HIS47 and the base forming residue HIS6 of human PLA2.

**Table 10:** Binding affinity (kJ/mol) and interactions of BR4 and BA with phospholipase A2 (1KQU)

Compound Name	Binding affinity (kJ/mol)	Amino acid and ligand (BR4/BA) interactions	Bond Distances (Å)	Category of interaction	Types of interaction
6-Phenyl-4(R)-(7-Phenyl-heptanoylamino)-hexanoic acid (BR4)	−33.89	LYS62[H…O]BR4	2.870	Hydrogen Bond;Electrostatic	Salt Bridge;Attractive Charge
		[Ca^+ 2^…O]BR4	4.024	Electrostatic	Attractive Charge
		BR4[NH…N]HIS47	1.829	Hydrogen Bond	Conventional Hydrogen Bond
		GLY29[NH…O]BR4	1.796	Hydrogen Bond	Conventional Hydrogen Bond
		GLY31[NH…O]BR4	2.374	Hydrogen Bond	Conventional Hydrogen Bond
		CYS28[H…O]BR4	2.601	Hydrogen Bond	Carbon Hydrogen Bond
		VAL30[H…O]BR4	2.388	Hydrogen Bond	Carbon Hydrogen Bond
		LYS62[H…O]BR4	2.941	Hydrogen Bond	Carbon Hydrogen Bond
		[Ca^+ 2^…O]BR4	2.767	Other	Metal-Acceptor
		[Ca^+ 2^…O]BR4	2.489	Other	Metal-Acceptor
		BR4[Pi…Pi]BR4	4.726	Hydrophobic	Pi-Pi T-shaped
		GLY29-VAL30[CON…BR4]	4.326	Hydrophobic	Amide-Pi Stacked
		BR4[Pi…Alkyl]LEU2	4.891	Hydrophobic	Pi-Alkyl
		BR4[Pi…Alkyl]ALA17	4.480	Hydrophobic	Pi-Alkyl
		BR4[Pi…Alkyl]ALA18	5.058	Hydrophobic	Pi-Alkyl
		BR4[Pi…Alkyl]LEU2	4.483	Hydrophobic	Pi-Alkyl
Betulinic acid (BA)	−41.00	GLY22[H…O]BA	2.493	Hydrogen Bond	Carbon Hydrogen Bond
		GLY29[H…O]BA	2.714	Hydrogen Bond	Carbon Hydrogen Bond
		BA[Pi…Sigma]PHE5	3.783	Hydrophobic	Pi-Sigma
		BA[Alkyl…Alkyl]LEU2	5.406	Hydrophobic	Alkyl
		BA[Alkyl…Alkyl]LEU2	4.322	Hydrophobic	Alkyl
		BA[Alkyl…Alkyl]ALA18	3.715	Hydrophobic	Alkyl
		BA[Alkyl…Alkyl]LEU2	3.813	Hydrophobic	Alkyl
		BA[Alkyl…Alkyl]ALA18	3.870	Hydrophobic	Alkyl
		BA[Alkyl…Alkyl]ALA18	3.892	Hydrophobic	Alkyl
		BA[Alkyl…Alkyl]LEU2	4.924	Hydrophobic	Alkyl
		LEU2[Alkyl…Alkyl]BA	3.962	Hydrophobic	Alkyl
		ALA17[Alkyl…Alkyl]BA	5.402	Hydrophobic	Alkyl
		ALA17[Alkyl…Alkyl]BA	5.356	Hydrophobic	Alkyl
		ALA18[Alkyl…Alkyl]BA	3.737	Hydrophobic	Alkyl
		PHE5[Pi…Alkyl]BA	5.290	Hydrophobic	Pi-Alkyl
		PHE5[Pi…Alkyl]BA	4.239	Hydrophobic	Pi-Alkyl
		HIS6[Pi…Alkyl]BA	5.133	Hydrophobic	Pi-Alkyl
		HIS6[Pi…Alkyl]BA	4.162	Hydrophobic	Pi-Alkyl
		HIS47[Pi…Alkyl]BA	5.074	Hydrophobic	Pi-Alkyl
		HIS47[Pil…Alkyl]BA	5.073	Hydrophobic	Pi-Alkyl
		TYR51[Pi…Alkyl]BA	4.321	Hydrophobic	Pi-Alkyl

On the other hand, the standard compound BR4 forms hydrogen bonding with LYS62, HIS47, GLY29, GLY31, CYS28, VAL30 and LYS62. Different types of hydrophobic interactions were also displayed by BR4 with GLY29-VAL30, LEU2 and ALA1. Moreover, BR4 exhibited attractive electrostatic interactions with Ca^+ 2^ ions.

### Binding affinity and in vitro inhibitory activity

A comparison was made between the binding affinities and in vitro inhibitory activities (IC_50_) of LY 311727, Manoalide, Indomethacin, Manoalogue, Aristolochic Acid and 1,1,1-Trifluoro-2-heptadecanone. The data has been presented in Table 11. Similar protocol described earlier was followed during performing docking of these compounds with PLA2 (1KQU). From the table it was clear that generally compounds with higher binding affinities also possess higher inhibitory activities. The binding affinities and reported PLA2 inhibitory concentration of BA are − 41.00 kJ/mol and ~ 2.5 μM for 30% & ~ 5 μM for 40% [5] inhibition which is consistent in accordance to our findings of binding affinity and in vitro inhibitory activity.

**Table 11:** Binding affinities (kJ/mol) and in vitro inhibitory activity (IC_50_) of different PLA2 inhibitors

Compound name	IC_50_ (μM) [86–88]	Binding affinity (kJ/mol)
LY 311727	< 1	−39.75
Manoalide	16	−39.33
Indomethacin	35	−36.82
Manoalogue	26	−34.31
Aristolochic Acid	40	−33.89
1,1,1-Trifluoro-2-heptadecanone	45	−27.20

## Conclusions

The acid dissociation constant (pKa), distribution coefficient (logD), partition coefficient (logP), aqueous solubility (logS), solvation free energy, dipole moment, polarizability, hyperpolarizability and different reactivity descriptors such as the chemical potential, electrophilicity, chemical hardness and chemical softness of betulinic acid have been calculated using MarvinSketch 15.6.29 and Gaussian 09 software. The calculated properties showed that betulinic acid has three species (one unionized and two ionized) over the pH range of 0.0 to 14.0 suggesting the unionized form is likely to exist in the acidic pH of the stomach however the betulinate form (ionized form) would be the predominant form in the alkaline pH of the intestine.

The logP, logD and logS values suggest that betulinic acid is non-polar and hydrophobic. This property would enable betulinic acid to pass through the lipid bilayer membrane of the cells. This hypothesis was corroborated by the results of HIA and permeability of betulinic acid through Caco-2 cell. It was found that BA has low skin permeability hence topical application BA as an anti-inflammatory agent can be achieved. Further, being lipophilic in nature betulinic acid would also exhibit positive CNS activity due to high permeability through BBB as evident from our in silico data.

The computational investigation of solvation free energy in different solvents explains the relative stability or reactivity of betulinic acid. Our theoretical calculation suggests that betulinic acid stabilized more in non-polar protic solvent due to hydrophobic interaction between the steroidal nucleus with the non-polar region of solvent and the electrostatic interaction between carboxyl group of betulinic acid with the polar portion of the solvent. Due to greater interaction with non-polar solvent the HOMO-LUMO gap becomes shorter as compared to the polar solvent (Table 5 and Fig. 11). It could also be inferred that it is this property which facilitates the hydrophobic interactions and formation of hydrogen bond of BA at the binding site cavity of PLA2.

BA was found to have 100% plasma protein binding and hence it could be deduced that it is unlikely to be eliminated promptly from the systemic circulation upon administration. Since BA acts as both inhibitor and substrate for P glycoprotein due to complex modulatory interactions with the Pgp [83], it is unlikely to be pumped out of the cells completely hence reducing the possibility of resistance mediated by efflux pump.

BA is likely to be metabolized by Cytochrome P450 3A4. However, it must be noted that it is an inhibitor for the same enzyme which could be overcome with the administration of Cytochrome P450 3A4 inducer. Since BA was also found to be an inhibitor of Cytochrome P450 2C9, it should not be used when a drug (which acts as a substrate for this enzyme) needs to be metabolized.

The toxicological study revealed that although betulinic acid is a mutagenic compound, it was noncarcinogenic in mice model. Moreover, molecular docking study revealed that betulinic acid interacts with GLY22 & GLY29 through hydrogen bond formation and LEU2, PHE5, HIS6, ALA17, ALA18, HIS47 and TYR51 through different types of hydrophobic interactions with a binding affinity of − 41.00 kJ/mol.

These computed molecular properties may assist to develop analytical method [46] to assay BA and the pharmacokinetic, toxicology and molecular docking study may provide a guide to synthesize betulinic acid derivatives with better pharmacokinetic & toxicological properties with potent phospholipase A2 inhibitory activity.

## Abbreviations

BA: Betulinic acid
BR4: 6-Phenyl-4(R)-(7-Phenyl-heptanoylamino)-hexanoic acid
DMSO: Dimethyl sulfoxide
HOMO: Highest occupied molecular orbital
LUMO: Lowest unoccupied molecular orbital
PDB: Protein Data Bank
PLA2: phospholipase A2
RMSD: Root mean square deviation
SMD: Solvation Model on Density
